# Donor nerve graft assessment for covering thumb nerve defects: a cadaveric study

**DOI:** 10.1186/s13018-020-01974-2

**Published:** 2020-10-06

**Authors:** Hamid Namazi, Ahmad Sobhani, Saeed Gholamzadeh, Amirreza Dehghanian, Fatemeh Dehghani Nazhvani

**Affiliations:** 1 Bone and Joint Diseases Research Center, Shiraz University of Medical Sciences, Shiraz, Iran; 2 Legal Medicine Research Center, Legal Medicine Organization, Tehran, Iran; 3 Trauma Research Center, Shiraz University of Medical Sciences, Shiraz, Iran

**Keywords:** Nerve allografts, Peripheral nerve reconstruction, Thumb nerve defect, Donor nerves, Anatomical zoning system

## Abstract

**Background:**

Even though several studies reported donor autologous nerve grafts for digital nerve defects, there is no report in the literature regarding acceptable graft for thumb nerves. The purpose of this study is to provide guidelines for autologous nerve graft selection by detecting similarities between thumb nerve zones and donor nerve with regard to the number of fascicles and cross-sectional area.

**Materials and methods:**

Five cadavers were used in this study. An anatomical zoning system was defined for thumb nerves (zones 1, 2, 3). Sural nerve (SN), medial antebrachial cutaneous nerve (MABCN), lateral antebrachial cutaneous nerve (LABCN), posterior interosseous nerve (PIN), and anterior interosseous nerve (AIN) were selected as donor nerve grafts. The number of fascicles and surface area (mm^2^) was defined.

**Results:**

The mean of the fascicle number in zone 1, zone 2, zone 3, AIN, PIN, LABCN, MABCN, and SN were 3.8, 4.7, 6.1, 2.2, 1.8, 4.5, 3.1, and 6.4, respectively. The mean of the surface area in zone 1, zone 2, zone 3, AIN, PIN, LABCN, MABCN, and SN were 2.19, 6.26, 4.04, 1.58, 0.71, 5.00, 3.01, and 8.06, respectively.

**Conclusions:**

LABCN is the best choice for all zones that has fascicular matching with all three zones of thumb nerves and caliber matching with zones 2 and 3. In zone 1, the best nerve graft is MABCN which has both suitable caliber and fascicle count.

## Introduction

Untreated digital nerve injury of the thumb can lead to significant sensory impairment. In cases of nerve gap, an autologous nerve graft is considered as the gold standard to bridge defects [1, 2]. So it is crucial to assess the available donor nerves for reconstructing thumb nerve defects. Several studies reported donor autologous nerve grafts for digital nerve defects, but there is no report in the literature regarding acceptable graft for thumb nerves [3–6].

Thumbs have four functional muscles that are mostly motor innervated by the lateral terminal branch of the median nerve and then by the deep branch of the ulnar nerve [7]. Generally, the median nerve divides into three common palmar digital nerves, which the first one trifurcates and two of them supply the sides of the thumb. Three variations had been reported by Jolley et al. in thumb-index finger sensory innervations in 1997 [8]. Type I with a rate of 69% has a radial thumb palmar digital nerve and a common palmar digital nerve that bifurcates at the first web space and innervates the ulnar side of the thumb and the radial side of the index finger [8]. Type II has a common thumb palmar digital nerve and a radial index finger digital nerve present in 6% of cases, and type III with a trifurcation of the median nerve has proper digital nerves to radial and ulnar thumb and radial index finger, existing in 25% [8]. In 1985, Hirasawa et al. reported the thumb digital nerve trifurcation into the main, median, and lateral branches before distal palmar thumb crease [9]. In 2002, Higgins et al. tried to cater a nerve graft selection guideline for the middle finger [10], but up to now, no survey for thumb has been done as the main finger for gripping.

Considering the importance of the thumb function in the quality of life and criticality of meticulous nerve repair, this study was designed to provide a guideline for autologous nerve graft selection in case of thumb nerve defects, emphasizing on thumb nerve zone similarities to donor nerve based on histological measurements. As near-matching fascicular patterns and caliber of stumps result in optimum functional recovery, fascicles count and surface area measurements were considered for the present histological assessment [11–13].

## Methods

**Fig. 1:**
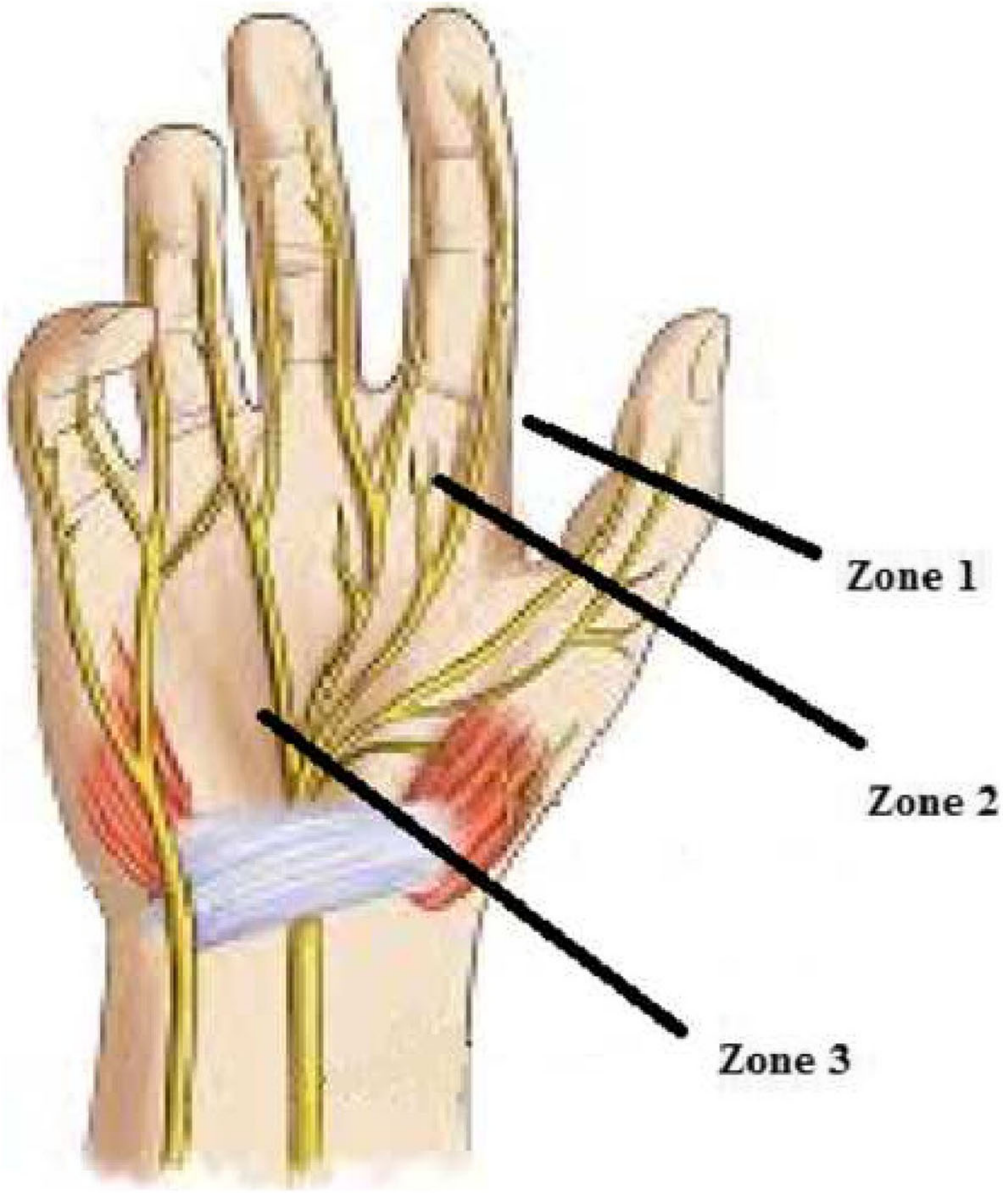
Schematic of thumb nerve zones. The figure illustrates the anatomical description of the palm, emphasizing on palmar digital nerves and zoning of the thumb. Zone 1 = distal to the interphalangeal joint. Zone 2 = proximal to the interphalangeal joint. Zone 3 = distal to the flexor retinaculum and proximal to the metacarpophalangeal joint

At fall 2019, twenty upper and lower limbs of five cadavers were used. At the palmar surface, median nerve ramification is obvious. Based on a defined anatomic zoning system (Fig. 1), zone 3 (distal to the flexor retinaculum and proximal to the metacarpophalangeal joint) consists of two proper palmar digital nerves branched out of the median nerve, while zone 2 (proximal to the interphalangeal joint) encompasses three branches that one of them travels deeply to the dorsal surface. And zone 1 (distal to the interphalangeal joint) contains two branches of the median nerve at the palmar surface. The specimens were harvested from the proximal of each zone to standardize the harvest of thumb nerves by the hand surgeon.

The sural nerve (SN), medial antebrachial cutaneous nerve (MABCN), lateral antebrachial cutaneous nerve (LABCN), posterior interosseous nerve (PIN), and anterior interosseous nerve (AIN) were selected as donor nerve grafts. The SN was harvested at the level of the lateral malleolus. The AIN was identified in the proximal border of the pronator quadrates and then the AIN was dissected and harvested at the branching point in the muscle. The PIN was identified at the midpoint of the pronator quadrates muscle belly and harvested by dorsal incision on the distal forearm before entering the wrist joint. The MABCN and LABCN were dissected at the elbow joint between the proximal and middle thirds of the forearm.

Harvested specimens were sectioned and stained by hematoxylin and eosin and examined through an Olympus microscope BX41 in a single-blind manner by the pathologist. Fascicle count and surface area measurements were done. Then data were analyzed using SPSS software, version 22 (SPSS Inc., Chicago, IL, USA). *T* test was performed to compare the variables, and *P* values less than 0.05 were considered significant meaning the specimens are not matched. *P* values more than 0.05 showed that samples were matched with no statistical differences and are appropriate for grafting.

## Results

**Fig. 2:**
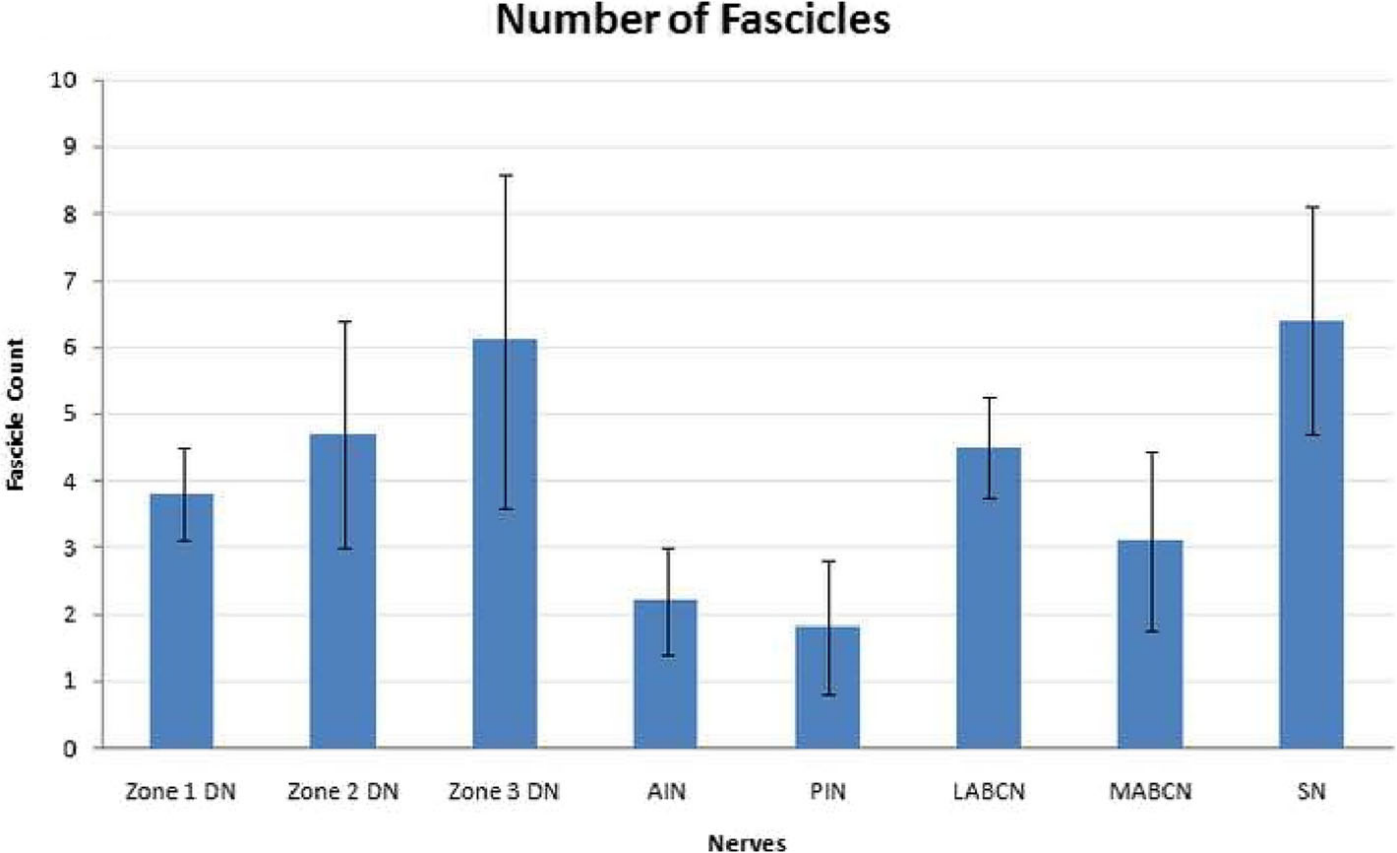
Fascicle counts in zones of digital nerves and donor nerve grafts. Bar chart showing the mean (SD) of the number of fascicles of different samples. DN, digital nerve; AIN, anterior interosseous nerve; PIN, posterior interosseous nerve; LABCN, lateral antebrachial cutaneous nerve; MABCN, medial antebrachial cutaneous nerve; SN, sural nerve

The demographic data of the used cadavers (ranging in age from 68 to 75 years) are mentioned in Table 1. The mean (SD) number of nerve fascicles of all specimens have been shown in Fig. 2. *T* test analysis revealed that as donor nerves, AIN and PIN fascicle counts differed significantly than all three zones of the thumb (*P* ≤ 0.001), while LABCN had non-significant differences with all three zones (*P* = 0.125, *P* = 0.776, and *P* = 0.151, respectively, for zone 1, zone 2, and zone 3). MABCN fascicle count differed significantly with zones 2 and 3 (*P* = 0.035 and *P* = 0.005, respectively) but showed a non-significant difference with zone 1 (*P* = 0.168). And SN fascicle count had significant differences with zones 1 and 2 (*P* = 0.001 and *P* = 0.012, respectively) but did not differ significantly with zone 3 (*P* = 0.059).

**Table 1:** Demographic data of the cadavers

Cadaver	Sex	Height	Weight	Birth location
1	M	160	72	Fars, Iran
2	M	172	78	Fars, Iran
3	M	168	83	Fars, Iran
4	M	175	86	Fars, Iran
5	M	164	69	Fars, Iran

**Fig. 3:**
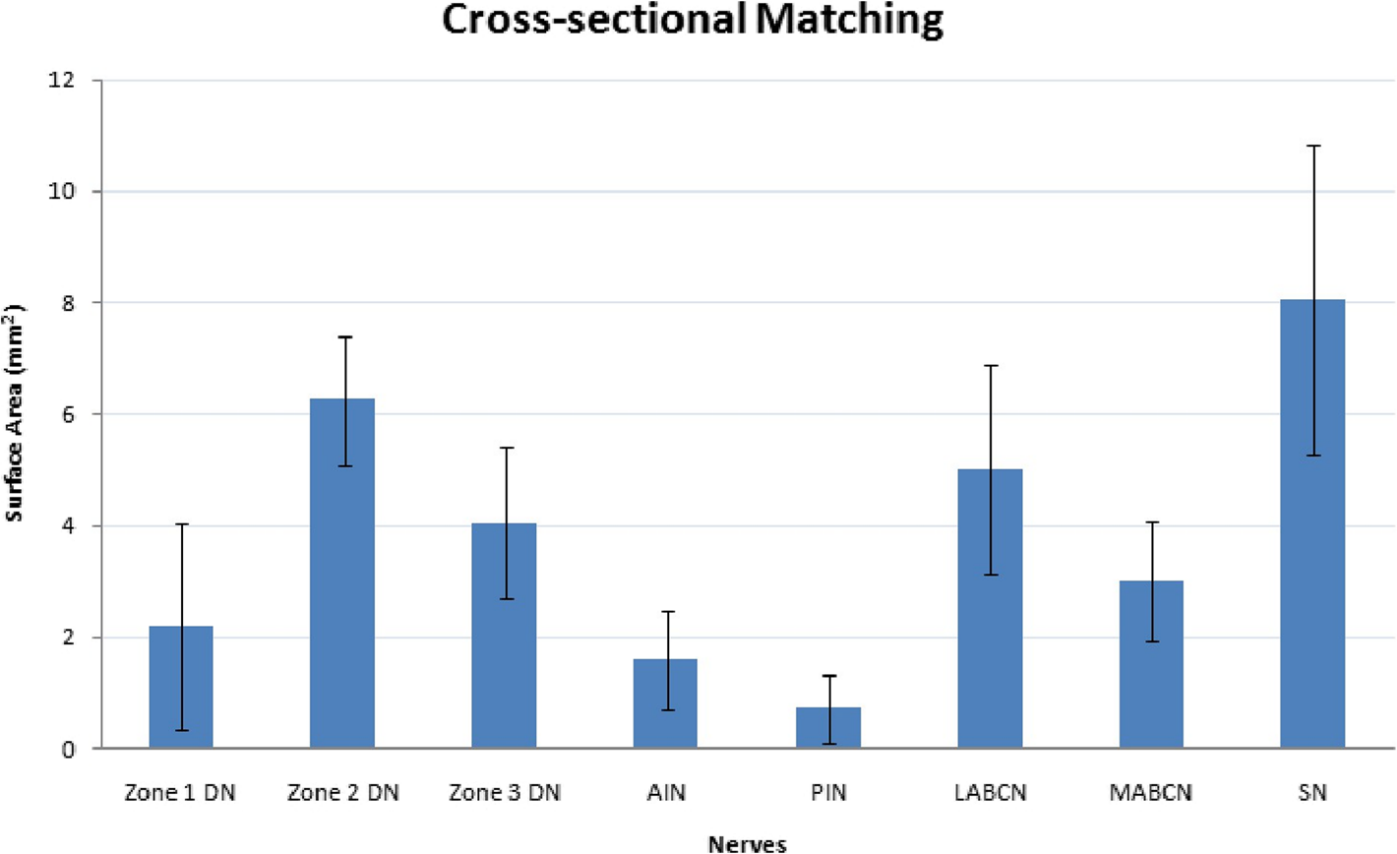
Cross-sectional assessment in zones of digital nerves and donor nerve grafts. Bar chart showing the mean (SD) of the surface area (mm^2^) of different samples. DN, digital nerve; AIN, anterior interosseous nerve; PIN, posterior interosseous nerve; LABCN, lateral antebrachial cutaneous nerve; MABCN, medial antebrachial cutaneous nerve; SN, sural nerve

The mean (SD) surface area (mm^2^) of cross-sectional matching in all nerve specimens is shown in Fig. 3. *T* test analysis showed that as donor nerves, just PIN surface area differed significantly than all three zones of thumb (*P* = 0.004 for zone 1 and *P* ≤ 0.001 for zones 2 and 3). AIN surface area differed significantly with zones 2 and 3 (*P* = 0,010 and *P* = 0.001, respectively) but had no significant difference with zone 1 (*P* = 0.489).

**Fig. 4:**
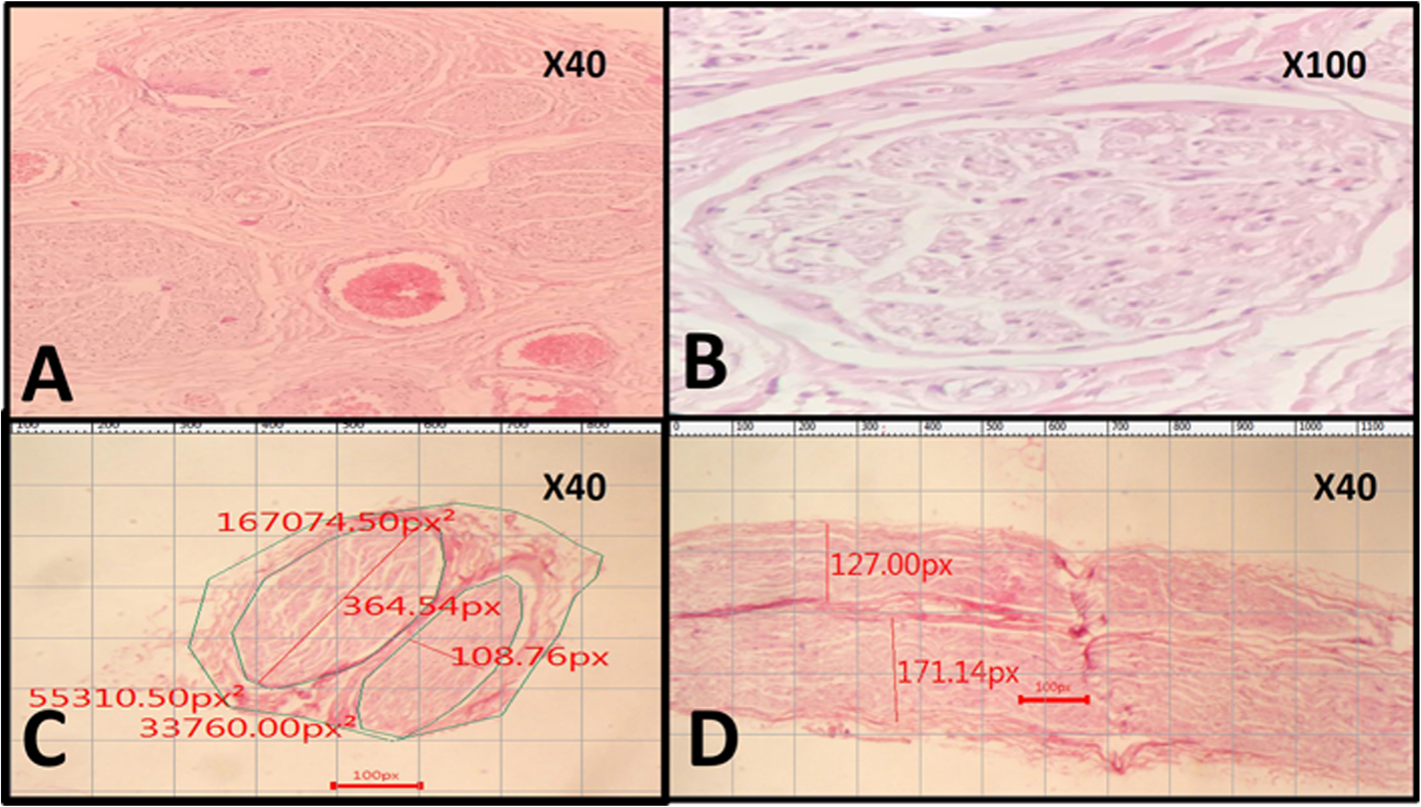
Histopathological sections of the nerves and the method of dimensions and surface area measurement. **a** Histological section of the medial antebrachial cutaneous nerve (MABCN) shows fascicles number and their arrangement. In this image, you can see 5 fascicles (H&E stain, × 40). **b** One fascicle (H&E, × 100). **c**, **d** Histological section of the lateral antebrachial cutaneous nerve (LABCN) cross-section (**c**) and longitudinal section (**d**), in which you can see two fascicles with their measurements including the maximum diameter of each fascicle, surface area of each fascicle, and total surface area of the nerve in pixels. The microscope camera’s software converts the measurements in to millimeters/millimeters^2^ (H&E stain, × 40)

MABCN surface area differed significantly with zone 3 (*P* = 0.006) but showed non-significant differences with zones 1 and 2 (*P* = 0.233 and *P* = 0.279, respectively). LABCN and SN surface areas had significant differences with zone 1 (*P* = 0.003 and *P* = 0.001, respectively), while they did not differ significantly with zones 2 and 3 (*P* = 0.648, *P* = 0.302 and *P* = 0.093, *P* = 0.053, respectively) (Fig. 4).

## Discussion

In cases of nerve defect, tension is possible at the repair site. When the injured nerve underwent a high tensile stress by simple microsurgical repair, the best technique for reconstruction is nerve graft or nerve transfer [14–17]. Although up to now, many techniques including conduits, veins, and allografts have been used to cover finger’s nerve defects, the best is to do autologous nerve graft [18–20]. Autografts are superior to the allografts because they are genetically identical with the recipient tissue. So, while allografts take longer time to incorporate into the recipient’s body, autografts decrease clinical failure.

Careful controlled electrolyte homeostasis of neurons makes action potentials and antegrade/retrograde axoplasmic transport of neurotransmitters [4]. Previous studies proved that the only significant elements for functional recovery are entering axons throughout the fascicles, which contain endoneurium [12, 13]. The number of fascicles of palmar digital nerves increases from proximal to distal level (from zone 3 to zone 1), but fascicle diameters are much smaller at the distal portion than the proximal. Also, this number is higher for nerves of the thumb and then the index fingers than the others, as the thumb has the highest fascicle count at proximal and distal levels [21]. The count of myelin nerve fibers normally decreases from proximal to distal level of palmar digital nerves, in which the number of these fibers is higher for the thumb, index, and long fingers and less for the ring and small fingers [21]. Thus, near-matching fascicular patterns of stumps can result in optimum axons sprouting throughout the recipient fascicles [11]. The diameter and cross-sectional area of nerves are important factors in nerve reconstruction. These measurements are nearly the same in palmar digital nerves of the thumb and other fingers [21]. However, for more precise technical success in nerve defect reconstruction, selecting the best cross-section (surface area), the number of fascicles and length match are clinically important to have the best functional outcome. A significantly smaller nerve graft in diameter than the proximal stump of the recipient nerve leads to fascicle lost and the possibility of neuroma formation. Due to more required time for regeneration, excessive length of the graft causes loss in numbers of regenerative fascicles of the nerve graft by increasing the risk of atrophy and fibrous ingrowth [11]. And smaller distal stump of the nerve graft than the distal recipient stump results in no regeneration in some fascicles of the distal recipient nerve end [10, 11]. These mismatchings act as a break in the axonal bilayer lipid membrane and result in irreversible apoptosis [4]. Also, less sutures are needed in caliber matching of two nerve stumps, resulting in less scar formation at the neurorraphy site and more nerve regeneration chance [10, 22]. This study presented that in fascicle counts, LABCN matches with all three zones of thumb digital nerve. while MABCN just matches with zone 1 and SN just matches with zone 3. In cross-sectional matching, MABCN matches with zones 1 and 2 and LABCN and SN match with zones 2 and 3. while AIN just matches with zone 1 of the thumb digital nerve. Thus, for zone 3, SN and LABCN are the best grafts with acceptable matching in caliber and fascicle count. For zone 2, although all three LABCN, MABCN, and SN nerves are suitable in caliber, just LABCN matches the best in fascicle count. And in zone 1, the best nerve graft is MABCN which matches suitable in caliber and either in fascicle count.

Another noticeable factor in selecting a nerve graft is the ease to harvest and yield sensory loss. Among the evaluated nerves for graft, AIN and PIN are technically difficult to harvest with a minimal apparent scar but yield in trivial sensory loss at the donor site. LABCN and MABCN are easier to harvest with less apparent scar but result in significant sensory loss, although the dermatome overlap minimizes that. SN supplies a great long and easy harvesting graft, but with a squalid scar site and sensory loss of lateral aspect of the leg and foot ankle that lessens by time [10].

Higgin’s assay introduced AIN, PIN, and MABCN as caliber matches and LABCN as fascicle count match for fingertip digital nerve (zone 1). And ignoring fascicle match, they suggested PIN as the best one due to ease of harvesting and minimal sensory deficit at the donor site [10]. In the present study, AIN and MABCN are caliber matches, while LABCN and MABCN are fascicle count matches for fingertip digital nerve of thumb (zone 1). Thus, considering all the criteria for selecting the best donor nerve as grafts, this study revealed that MABCN is the best match for zone 1 in caliber and fascicle count.

For zones 2 and 3 of the middle finger, Higgin’s assay suggested LABCN, especially for zone 3 with equal fascicle count. In their point of view, LABCN had minor non-significant differences in caliber and fascicle count and either easy to harvest and negligible consequent sensory deficit because of the dermatome overlap by sensory branches of the radial nerve [10]. This time, although all three MABCN, LABCN, and SN are caliber matches for zone 2, LABCN is the best choice due to its fascicle count matching.

Finally, Higgins et al. introduce SN as the best match for zone 4 of their assay, although it has fewer fascicles and smaller caliber [10]. Here, in the same anatomical level, SN and LABCN match best with zone 3 of the thumb in both fascicle count and caliber. In addition to the surface area, the cross-sectional shape matching is also important. As SN is basically a flap [11], it seems to be more appropriate for zone 3 of the thumb digital nerve, which has either a flap shape.

LABCN nerve graft for fingers’ nerves was firstly done by McFarlane and Mayer in 1976 that 11 of 13 patients showed excellent two-point discrimination between 7 and 20 mm. They also concluded that LABCN has a good length to graft and is easy to obtain [23]. Tank et al. showed that LABCN has more similarity to the fingers’ nerves in the fascicle pattern [12]. Also, some clinical trials such as Schonauer et al., Pilanci et al., and Unal et al.’s works elucidated that more sensory regaining chance at recipient occurred by LABCN as interposed graft and encouraged by the advantage of LABCN uptake from the non-resting area of distal lateral of forearm that makes no critical problem in the patient [24–26].

Since Higgins et al. tried to cater a nerve graft selection guideline for the middle finger, the strong point of the present study is introducing a new practical guideline for thumb nerve defects as the main finger for gripping and the importance of its function in quality of life. But the disadvantages were its cadaveric modality and the small sample size of the study. Thus, the effectiveness of this guideline needs to be confirmed by more investigations on patients and correlated with clinical results. Also, it must be considered that axon count can demonstrate more precise results than fascicle count because some nerves have more fascicles with less axon fibers. However, combining such clinical trials with optimizing nerve repair techniques and drugs can be helpful to gain more valuable clinical results [27].

Considering the donor nerve grafts matching with thumb nerve zones in fascicle count and caliber (Table 1), LABCN is the best choice for all zones, which has fascicular matching with all three zones of thumb nerves and caliber matching with zones 2 and 3. This is also the consensus recommendation of literature [10, 28], as despite the notable donor site sensory deficit [10], its harvesting does not make disability and discomfort [28]. After that, SN and MABCN with more caliber matching are good choices. However, in addition to double preparation and anesthesia for SN harvesting than those of the upper limb, its high affinity to form sequelae and symptomatic painful neuroma must be considered [11, 28].

## Conclusion

LABCN is the best choice for all zones as it has fascicular matching with all three zones of thumb nerves and caliber matching with zones 2 and 3. In zone 1, the best nerve graft is MABCN which has both suitable caliber and fascicle count.

## Data Availability

The datasets used and/or analyzed during the current study are available from the corresponding author on reasonable request.
